# Functional pancreatic neuroendocrine tumors predominantly presenting with hypercalcemia: a case report

**DOI:** 10.3389/fonc.2025.1693096

**Published:** 2026-01-05

**Authors:** Jieting Duan, Yanping Zhu, Yan Wang, Xiang Kui, Yingmei Tang

**Affiliations:** 1 Department of Gastroenterology, The Second Affiliated Hospital of Kunming Medical University, Kunming, Yunnan, China; 2 Department of Pathology, The Second Affiliated Hospital of Kunming Medical University, Kunming, Yunnan, China

**Keywords:** functional pancreatic neuroendocrine tumors, hypercalcemia, parathyroid hormone-related protein, parathyroid hormone, continuous renal replacement therapy

## Abstract

Functional pancreatic neuroendocrine tumors (F-PNETs) that secrete parathyroid hormone-related protein (PTHrP) and cause hypercalcemia are extremely rare. This report details a 10-year course of a patient with pancreatic neuroendocrine tumor (PNET) and recurrent life-threatening hypercalcemia. Moreover, it comprehensively describes the evolution and management of hypercalcemia in this case, aiming to offer valuable insights for clinical diagnosis and treatment.

## 1 Introduction

Pancreatic neuroendocrine tumors (PNETs) are a rare type of neoplasm originating from the pancreatic endocrine tissues (1). Based on the hormone secretion and associated symptoms, PNETs are classified into functional (secretory) or nonfunctional tumors. The majority of PNETs are nonfunctional, accounting for 50%–70% (2). Insulinomas are the most prevalent among functional PNETs (F-PNETs), followed by gastrinomas, glucagonomas, VIPomas, somatostatinomas, carcinoid syndrome-related PNETs, and other rare subtypes (3, 4). PNETs may secrete parathyroid hormone-related protein (PTHrP), leading to hypercalcemia associated with malignancy (5, 6). Nevertheless, the hypercalcemia mediated by PTHrP in PNETs is exceptionally rare, which typically portends an unfavorable prognosis (7). This report presents a case of F-PNETs potentially secreting PTHrP, which primarily manifests as hypercalcemia. Furthermore, it analyzes the etiologies of hypercalcemia, explores effective calcium-lowering strategies, and discusses therapeutic challenges related to subsequent renal and cardiac failure, aiming to enhance the clinical awareness.

## 2 Case presentation

### 2.1 Discovery of tumors and surgical resection

A pancreatic mass was incidentally identified in an asymptomatic 64-year-old male during a health examination in July 2015. His past medical history includes an acute myocardial infarction (AMI) in 2017, treated with percutaneous coronary intervention (PCI) and stent implantation, Following the procedure, he was prescribed a regimen of aspirin, ticagrelor, and rosuvastatin. The patient denied any history of smoking or alcohol consumption,and his family history is unremarkable. The mass was subsequently identified as a PNET via endoscopic ultrasound-guided fine-needle aspiration (EUS-FNA) in November 2015. A CT scan revealed a 5.7 cm × 4.6 cm mass at the pancreatic tail and splenic hilum, suggesting a pancreatic malignancy with splenic vein invasion. In March 2016, the patient underwent distal subtotal pancreatectomy (pancreatic tail), splenectomy, and retroperitoneal tumor resection without receiving adjuvant therapy. Subsequently, he was confirmed to have a G2 NET (Ki-67: focal maximum of 15%) by histopathology.

### 2.2 Tumor progression and antiproliferative therapy

**Figure 1:**
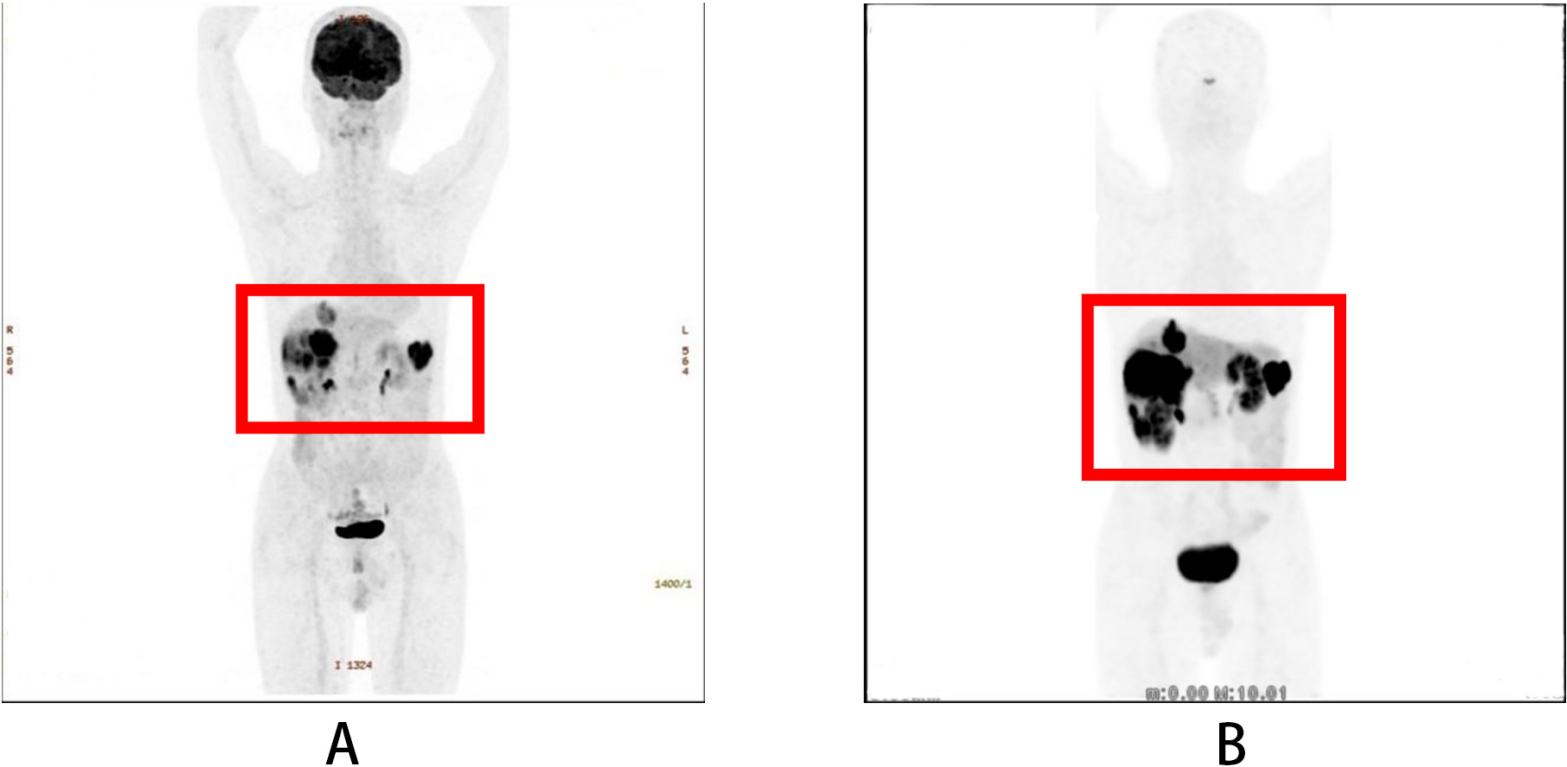
PET/CT (February 2023): **(A)** (^18^F-FDG) and **(B)** (^68^Ga-DOTANOC): The regions of interest (ROIs) are outlined in red, demonstrating: (1) an FDG-avid and somatostatin receptor (SSTR)-positive nodule in the pancreatic stump, suggesting recurrence; (2) peritoneal implants located in the gastric greater curvature and gallbladder regions, demonstrating intense FDG uptake and SSTR expression, with invasion of the gastric fundus; and (3) multiple hypermetabolic, SSTR-positive liver metastases.

**Figure 2:**
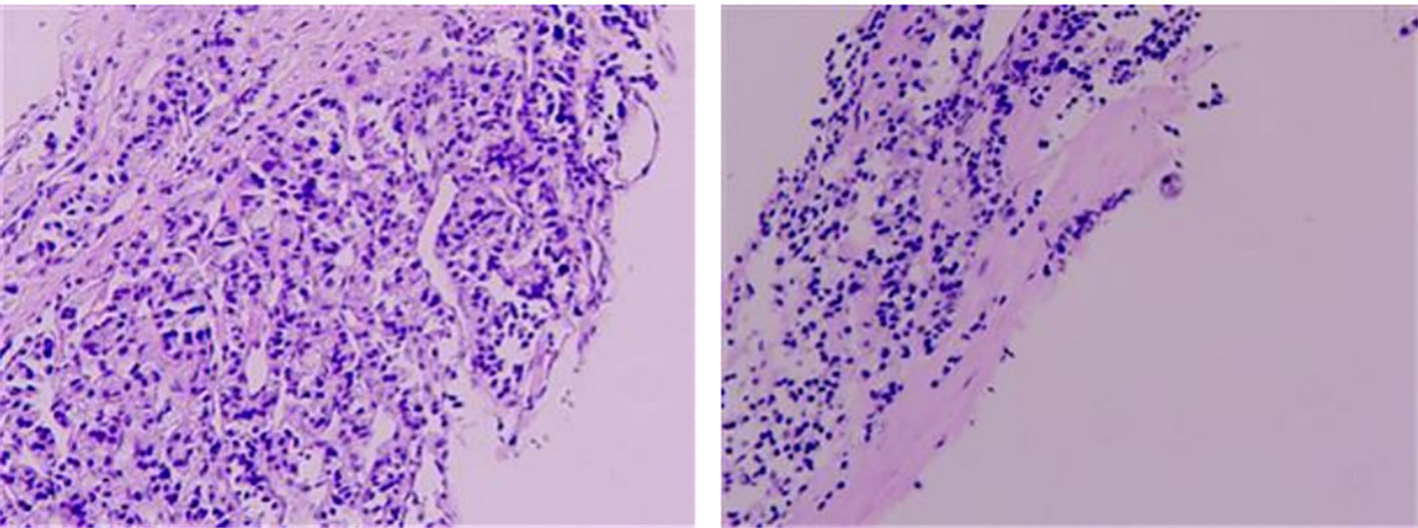
Liver biopsy (H&E stain, ×100). Tumor cells showed CK( +), CD56( +), Syn( +), CgA( +), TTF-1( −), PAX-8( −), and CDX-2( −), with focal heterogeneous P53( +), retained Rb expression, Ki-67 10% (hotspot), and mitotic count 1/2mm^2^.

**Figure 3:**
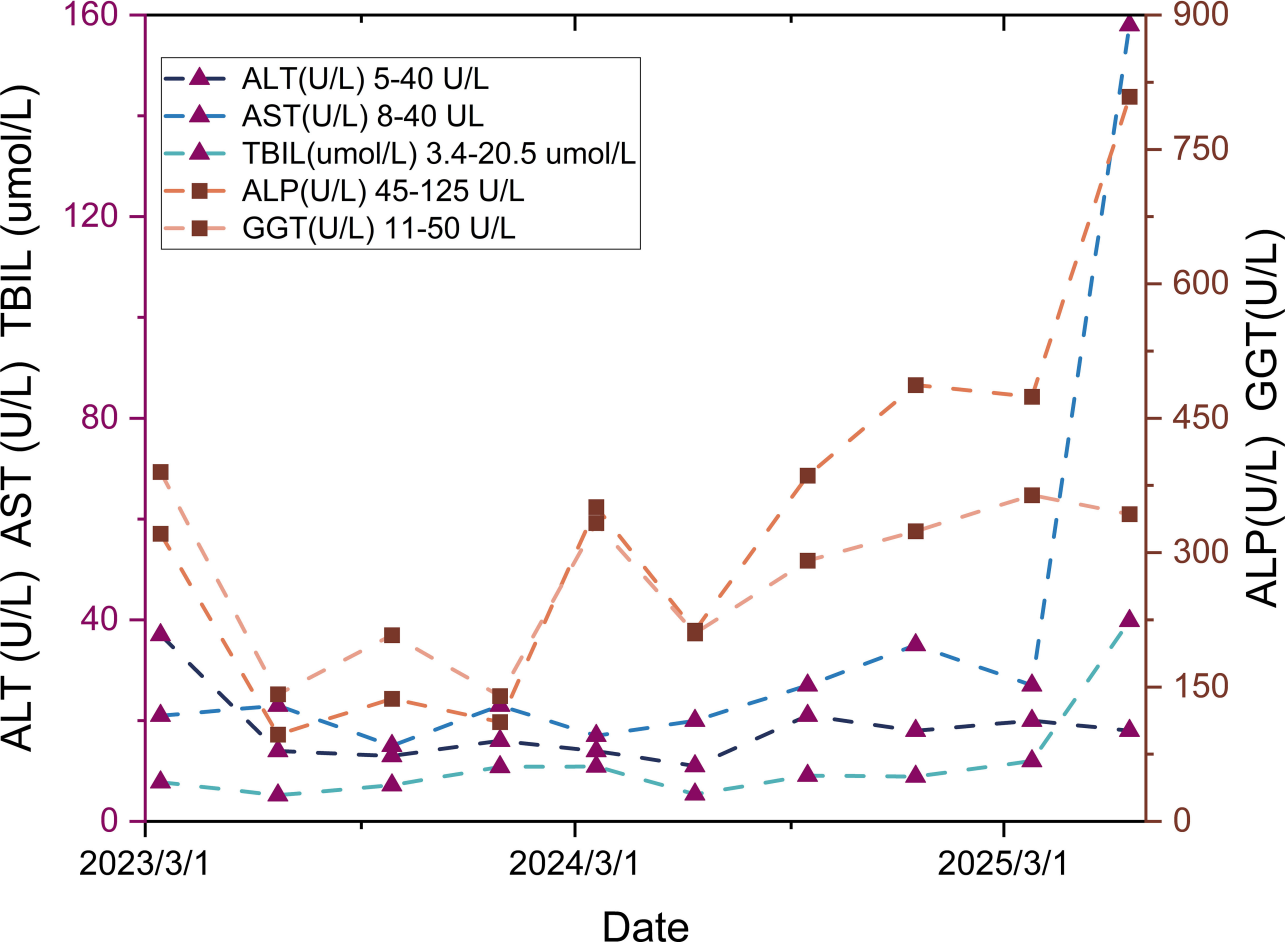
Liver function trend: Gradual elevation in alkaline phosphatase (ALP) and gamma-glutamyl transferase (GGT) levels is observed, whereas aspartate aminotransferase (AST), alanine aminotransferase (ALT), and total bilirubin(TBIL) remain stable.

**Figure 4:**
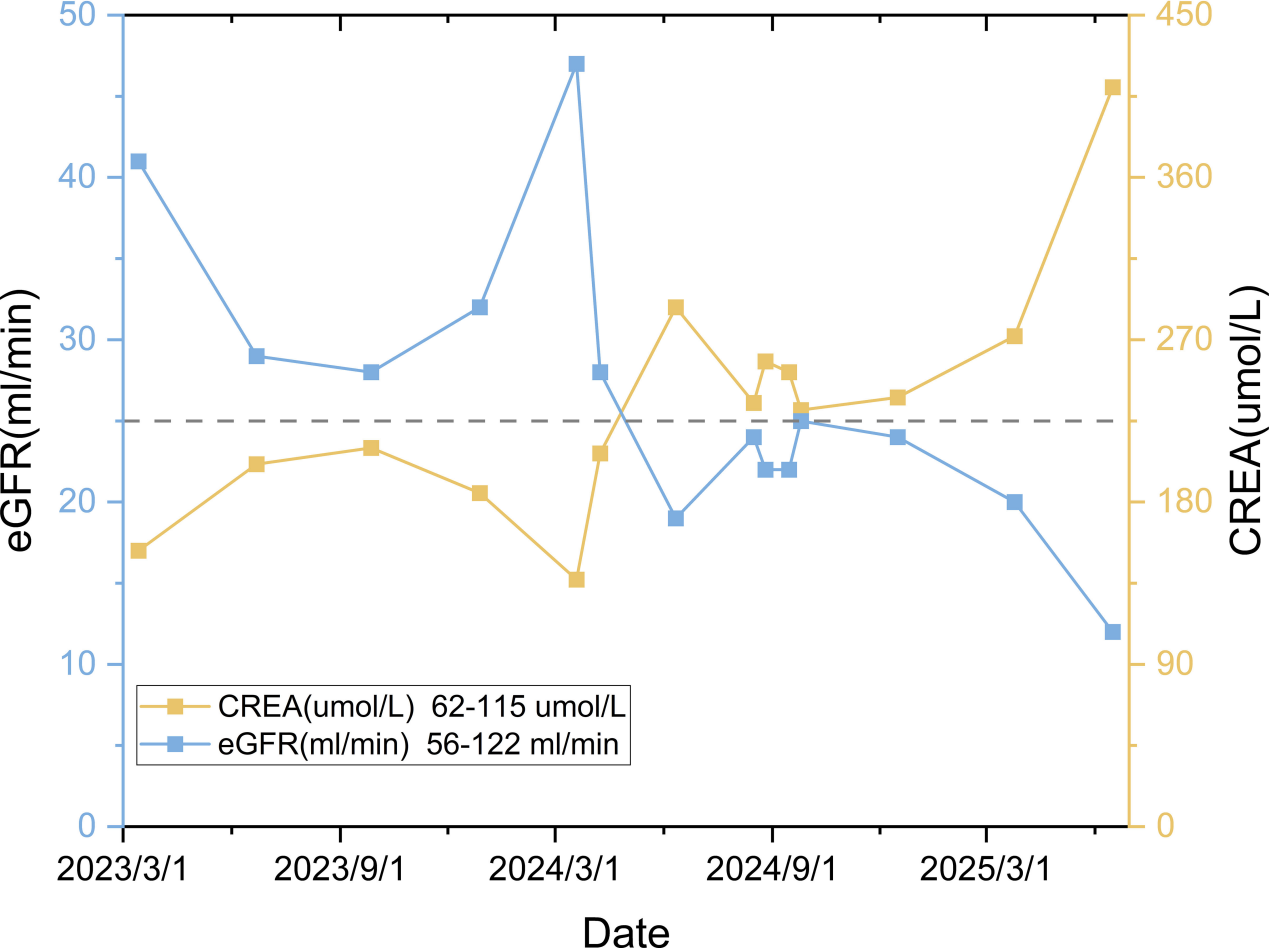
Renal function trend: Serum creatinine levels fluctuate in correspondence with renal replacement therapies, including but not limited to hemodialysis.

**Figure 5:**
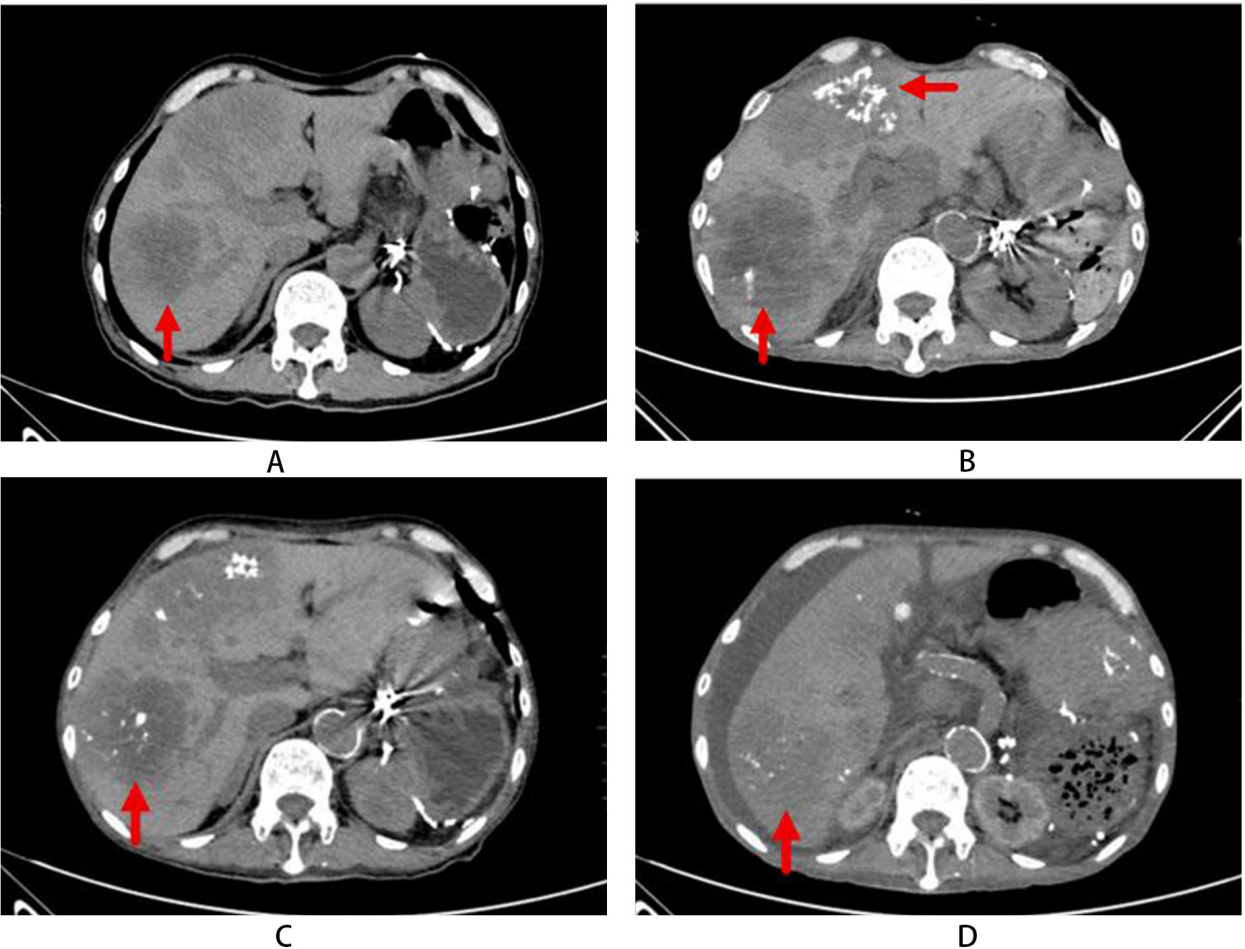
**(A)** (Mar 2023): The liver appears enlarged with preserved overall morphology. Multiple patchy and mass-like hypodense lesions are observed, consistent with diffuse hepatic metastases. **(B)** (Mar 2024): Follow-up CT post-TAE of hepatic metastases shows an increase in the size of target lesions compared with the prior study. **(C)** (Jul 2024): Subsequent CT post-TAE demonstrates that the hepatic metastatic lesions remain largely stable in size and appearance. **(D)** (May 2025): Further follow-up CT post-TAE indicates overall stability of the hepatic metastases, with no significant interval change.

Multiple hepatic lesions, with the largest measuring 4.0 cm × 2.9 cm, were detected in February 2017, suggesting metastases. Everolimus (10 mg daily) was initiated in March 2019 and continued for four years until February 2023, when it was discontinued due to the onset of secondary type 1 diabetes mellitus. Thereafter, the patient maintained well-controlled blood glucose levels with regular subcutaneous insulin injections. In November 2022, magnetic resonance imaging revealed an increase in the size of hepatic lesions (largest size: 5.6 cm × 5.0 cm). In February 2023, PET/CT with ^18^F-fluorodeoxyglucose (FDG) and ^68^Ga-DOTANOC (**Figure 1**) demonstrated the following signs: (1) the FDG-avid, somatostatin receptor (SSTR)-positive nodule in pancreatic stump, suggesting recurrence; (2) the peritoneal implants (gastric greater curvature/gallbladder regions) with intense FDG uptake and SSTR expression, invading the gastric fundus; and (3) multiple hypermetabolic, SSTR-positive liver metastases. The pathology of liver biopsy was shown in **Figure 2**. Tumor cells showed CK( +), CD56( +), Syn( +), CgA( +), TTF-1( −), PAX-8( −), and CDX-2( −), with focal heterogeneous P53( +), retained Rb expression, Ki-67 10% (hotspot), and mitotic count 1/2mm^2^. Findings confirmed well-differentiated neuroendocrine tumor (NET). A grade 2 transarterial embolization (TAE) was performed on March 2, 2023, followed by the initiation of long-acting octreotide (Sandostatin LAR^®^ 40 mg every 4 weeks) from March 14, 2023. The partial laboratory test results from the patient’s first visit to our hospital (March 2023) are shown in **Table 1**. Hepatic and Renal Function: The dynamic changes in hepatic and renal parameters are presented in **Figures 3**, **4**. A 12-lead electrocardiogram (ECG) demonstrated sinus bradycardia with a heart rate of 55 beats per minute. Thyroid and parathyroid ultrasonography revealed no sonographic evidence of parathyroid gabnormalities. A small, solid nodule was noted in the mid-portion of the left thyroid lobe, classified as C-TI-RADS 3 (probably benign, most suggestive of nodular goiter). No abnormal lymph nodes were identified in the bilateral cervical regions. A CT scan in March 2023 was detailed in **Figure 5A**.

**Table 1:** Laboratory findings from initial presentation to our hospital (March 2023).

Laboratory parameter	Value	Reference range	Laboratory parameter	Value	Reference range
Hemoglobin (HGB)	114 g/L	130–175 g/L	Calcitonin (CT)	>2000.00 pg/mL	<9.52 pg/mL
HbA1c (NGSP)	6.3%	4.6-6.5%	Parathyroid hormone (PTH)	5.17 pg/mL	15.00-65.00 pg/mL
Biochemical parameters			Osteocalcin (N-MID)	51.54 ng/mL	6.00-24.66 ng/mL
Serum calcium(Ca^2+^)	3.49 mmol/L	2.11-2.52 mmol/L	β-crosslaps (β-CTX)	2311.00 pg/mL	43.00-783.00pg/mL
Serum phosphorus(P)	0.85 mmol/L	0.85-1.51 mmol/L	25-hydroxy vitamin D	45.7 nmol/L	75.00-250.00 nmol/L
Alanine aminotransferase(ALT)	37 U/L	5–40 U/L	Thyroid function		
Aspartate aminotransferase(AST)	21 U/L	8–40 U/L	Thyroid-stimulating hormone (TSH)	0.19 mIU/L	0.27-4.20 mIU/L
Alkaline phosphatase(ALP)	321 U/L	45–125 U/L	Triiodothyronine (T3)	0.66 ng/mL	0.8-1.9 ng/mL
Gamma-glutamyl transferase(GGT)	390 U/L	11–50 U/L	Free T3 (FT3)	3.07 Pmol/L	3.5-7.0 Pmol/L
Total bilirubin(TBIL)	7.8 umol/L	3.4-20.5umol/L	Thyroxine (T4)	54.52 μg/L	50-130μg/L
Creatinine(CREA)	153 umol/L	62-115umol/L	Free T4 (FT4)	10.69 Pmol/L	10–22 Pmol/L
Estimated glomerular filtration rate(eGFR)	41 ml/min	56-122ml/min	Reverse T3 (RT3)	1.26 nmol/L	0.45-0.98 nmol/L
Bone metabolism			Thyrotropin receptor antibodies(TRAb)	<0.8 IU/L	0-1.75 IU/L

### 2.3 Recurrent hypercalcemic crisis, hyperkalemia, and renal impairment

From March 2023 to February 2024, recurrent abdominal distension/anorexia with persistent hypercalcemia/hyperkalemia suggested stepwise therapy: taking octreotide LAR (from 30 mg to 40 mg every 4 weeks), salmon calcitonin, and sodium zirconium cyclosilicate. The patient’s serum potassium returned to normal, but hypercalcemia still persisted despite the administration of zoledronic acid and methylprednisolone, as well as 11 sessions of hemodialysis. On March 15, 2024, the patient had altered mental status [calcium: 4.16 mmol/L(2.11-2.52 mmol/L); creatinine: 196 μmol/L(62-115μmol/L)]. The patient took continuous renal replacement therapy (CRRT) for 72 hours, resulting in normalization of calcium levels and improvement of symptoms. Combined liver-kidney transplantation was recommended but declined by the patient. A CT scan in March 2024 indicated the progression of disease (**Figure 5B**). Subsequent treatments included octreotide LAR (30 mg every 10 days), denosumab (150 mg initially, then 60 mg every 3 months) and intermittent CRRT to maintain normocalcemia (1.5 mmol/L transiently), albeit with persistent renal dysfunction.

### 2.4 Tumor stabilization and cardiac failure

During 6-month treatment with octreotide LAR (30 mg every 10 days), the patient’s calcium levels normalized but the creatinine rose progressively, accompanied by persistent hyperkalemia. Furthermore, the tumors retained stable (**Figure 5C**, as the pancreatic head-neck tumors, left upper abdominal metastases, and hepatic metastases all demonstrated stability without significant interval changes. The metastatic lesions within the anterior abdominal wall showed mild regression compared to prior imaging findings. In July 2024, the patient presented with bilateral lower limb edema, oliguria, and exertional dyspnea. Cardiac markers covered 0.328 ng/mL(<0.014 ng/mL) of troponin T, and >35,000 pg/mL(0–125 pg/mL) of PRO-BNP. Echocardiography revealed a severely reduced left ventricular ejection fraction of 35% (normal range: 49-79%), abnormalities of regional wall motion, and moderate mitral/tricuspid regurgitation. It also showed the morphologically normal mitral, tricuspid, and pulmonary valves with preserved mobility. Intensified octreotide therapy was taken to control tumors, but progressive renal dysfunction and new-onset cardiac failure required medical management and regular hemodialysis. The edema showed transient improvement during treatment.

### 2.5 Unexplained dyspnea

In April 2025, the patient experienced paroxysmal dyspnea (10–30 sec/episode; >10 episodes/day) with a sense of impending doom, so the etiologies of acute heart failure, asthma, diabetic ketoacidosis, and psychology were ruled out. The symptoms resolved after 12 hours of continuous somatostatin infusion and completely subsided within 72 hours.

A CT scan in May 2025 showed a reduction in tumor burden (**Figure 5D**), but the patient developed cachexia and succumbed to multiple organ failure on June 16, 2025.

## 3 Discussion

### 3.1 Etiology of hypercalcemia

Approximately 90% of hypercalcemia cases stem from classic humoral hypercalcemia of malignancy (HHM) or hyperparathyroidism (HPT). Both HHM and HPT are characterized by the increased osteoclast-mediated bone resorption driven by the following humoral factors: parathyroid hormone (PTH) in HPT and tumor-derived PTHrP in HHM (8). HHM is typically associated with squamous cell carcinomas such as esophageal and pulmonary cancers, along with renal cell carcinoma and breast cancers. Nevertheless, sporadic cases of HHM secondary to neuroendocrine tumors (NETs) have been recorded.

Two distinct forms of hypercalcemia may arise in metastatic gastroenteropancreatic neuroendocrine tumors (GEP-NETs): osteolytic localized hypercalcemia and HHM. The former arises from the direct activation of osteoclasts by metastatic tumor cells, whereas the latter stems from tumor hypersecretion of PTHrP into systemic circulation (9–11). Additionally, ectopic PTH secretion by NETs or carcinomas leads to severe HPT, which has been noted in sporadic case reports (12–14). Notably, patients with primary pancreatic, thymic, or pulmonary NETs manifested as hypercalcemia and elevated PTH require timely evaluation for primary hyperparathyroidism (PHPT) (15).

The patient presented with refractory hypercalcemia. Given the unavailability of PTHrP assay, this study conducted a multifactorial etiological analysis, and ultimately concluded that tumor-derived PTHrP hypersecretion was the most likely underlying mechanism.

**Figure 6:**
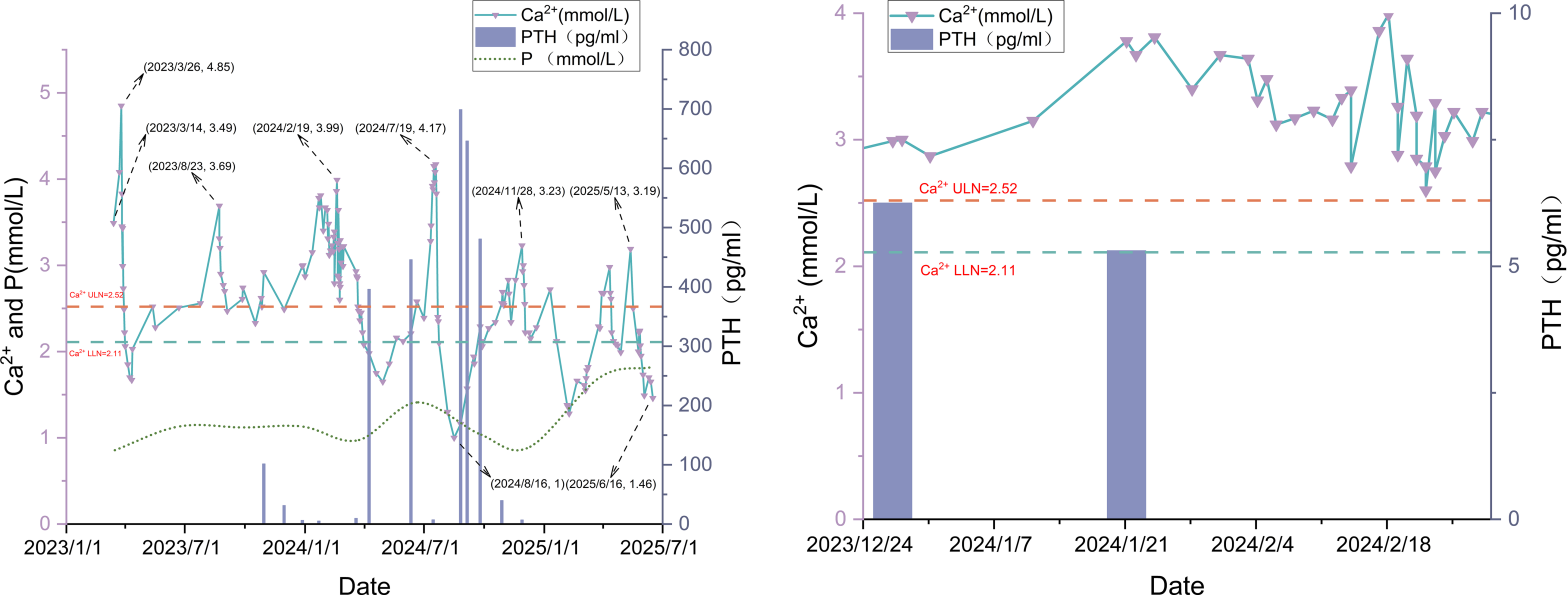
Trends of serum calcium, phosphorus and PTH levels. **Figure 6** Close-up: December 2023 – March 2024.

**Figure 7:**
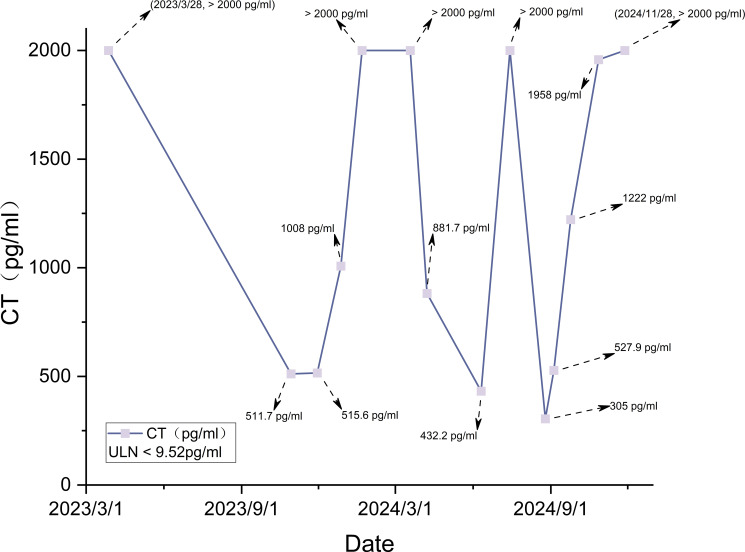
Calcitonin (CT) trend.

**Figure 8:**
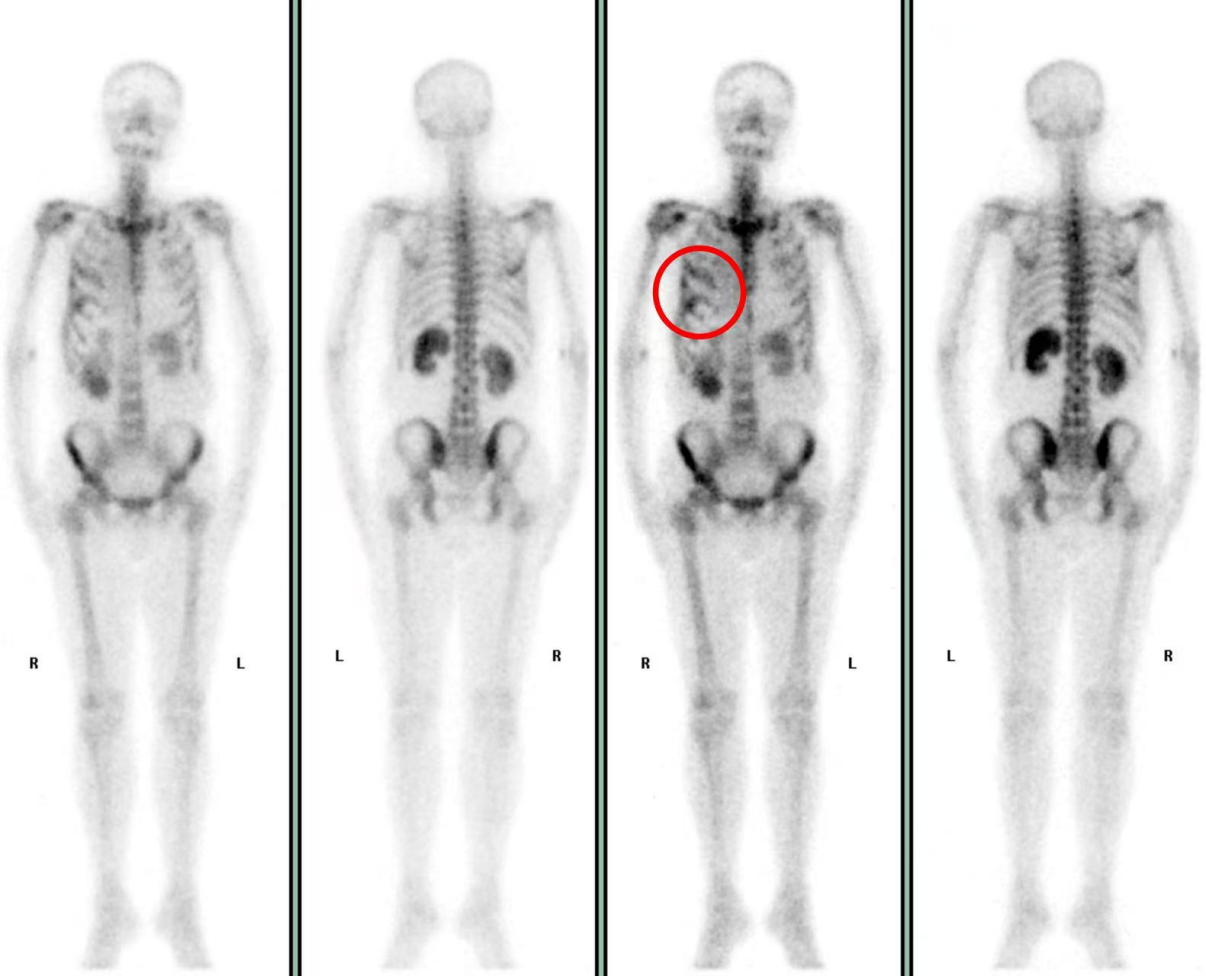
Whole-body bone scintigraphy (March 31, 2023): Abnormally increased metabolic activity is observed in the right 4th to 6th ribs, as indicated by the red circles.

1. PHPT was characterized by elevated PTH, which induced hypercalcemia and hypophosphatemia through multifactorial mechanisms. In this patient, the dynamic changes in PTH, serum calcium, phosphorus, and calcitonin levels (**Figures 6**, **7**) demonstrated an intact calcium–PTH negative feedback loop. In view of the unremarkable parathyroid ultrasonography and normal phosphatemia, these results explicitly ruled out both PHPT and tumor-mediated ectopic PTH secretion (16, 17).
2. Whole-body bone scintigraphy revealed increased localized osteoblastic activity in the 4th-6th right ribs (**Figure 8**), suggesting osteolytic metastases. Pathologically augmented bone resorption induced by direct cellular interactions between tumor cells and osteoclasts may constitute one of the etiologies underlying hypercalcemia.
3. Concurrent chronic kidney impairment decreased renal calcium excretion, representing another contributing factor to hypercalcemia.
4. Multiple case reports have demonstrated that PTH is concomitantly suppressed during PTHrP elevation (18), which confirms that PTHrP inhibits endogenous PTH secretion. This is manifested as low/undetectable PTH levels in PTHrP-mediated hypercalcemia. The patient presented with inconsistent calcium-PTH kinetics (rising calcium levels with suppressed PTH), indicating hypercalcemia driven by PTHrP (19). Treatment-induced reduction in PTHrP activity led to calcium normalization, accompanied by the reciprocal PTH elevation (**Figure 6**), which further validated the mechanism. The etiology of elevated PTH level was systematically evaluated. Primary hyperparathyroidism was definitively ruled out based on the inverse correlation between serum calcium and PTH levels, along with repeatedly negative parathyroid scintigraphy findings. Although renal insufficiency can theoretically lead to hypocalcemia and hyperphosphatemia, thereby stimulating PTH secretion and elevating PTH levels, in this patient, renal dysfunction progressed gradually, with no acute deterioration coinciding with the PTH elevation. Therefore, renal insufficiency was not considered the primary cause of the elevated PTH. The observed PTH elevation can be mechanistically attributed to three principal factors: (1) hypocalcemia reduces activation of the calcium-sensing receptor (CaSR) on parathyroid chief cells, thereby relieving its tonic inhibition on PTH secretion (19–22); (2) acute hypocalcemia triggers rapid exocytosis of pre-synthesized PTH from secretory granules, elevating circulating PTH levels within minutes; and (3) sustained hypocalcemia promotes intracellular processing of PTH for storage and subsequent secretion. The resulting fluctuations in PTH levels ultimately reflects the antagonistic effect of tumor-induced hypercalcemia, which intermittently suppresses PTH secretion through negative feedback, producing a cyclical pattern.

In conclusion, this case most likely represented a PTHrP secretion by F-PNET, with hypercalcemia as its primary manifestation (19, 23–25).

In 1978, PTHrP was identified as a tumor-derived secretory factor that mediates malignancy-associated hypercalcemia (7), which can be detectable in certain solid tumors such as lung carcinoma and renal cell carcinoma. Nevertheless, PTHrP secretion by GEP-NETs has rarely been documented.

PTHrP secretion by PNETs shows uniform gender distribution, which is consistent with the general epidemiology of PNETs (26). Hypercalcemia typically emerges months to years after PNET diagnosis, whereas a minority of patients have hypercalcemia when diagnosed (18, 27).

PTHrP is structurally homologous to PTH, which can activate the PTH type 1 receptor (28), and induce comparable physiological effects. These effects include the stimulation of osteoclast-mediated bone resorption and the enhancement of calcium reabsorption in the renal distal tubules, which in turn hinders renal calcium excretion.

Hepatic metastasis represents a pivotal factor in the development of hypercalcemia. Most reported cases of PTHrP secretion by NETs have liver metastases (29). As early as 6 years prior to the onset of hypercalcemia, the immunohistochemical verification of PTHrP in metastatic lesions demonstrates prolonged subclinical secretion (30). The progression of tumor dedifferentiation and increased tumor burden ultimately result in sufficient circulating PTHrP to trigger hypercalcemia.

### 3.2 Diagnosis

Histopathology remains the gold standard for diagnosing neuroendocrine tumors (NETs). NETs are broadly classified into well-differentiated NETs and poorly differentiated neuroendocrine carcinomas (NECs). Well-differentiated NETs are further stratified into grades G1, G2, and G3 based on mitotic count and the Ki-67 proliferation index. Endoscopic ultrasound-guided fine-needle biopsy (EUS-FNB) has emerged as the preferred diagnostic tool for pancreatic NETs (P-NETs). Recent studies indicate that, compared to conventional fine-needle aspiration (FNA), EUS-FNB yields larger, architecturally preserved tissue samples, which is crucial for accurate tumor grading, particularly in assessing the Ki-67 index. Therefore, EUS-FNB is recommended as the first-line biopsy approach for suspected P-NETs (31–36). Nevertheless, further randomized studies are warranted to validate these findings (37, 38).

### 3.3 Therapeutic management

PTHrP-mediated hypercalcemia in PNETs poses therapeutic challenges typically. The goals of therapy involve maintaining normocalcemia, tumor stabilization or regression, and ultimately extending progression-free survival.

#### 3.3.1 Cytoreductive therapy

Complete surgical resection of primary and metastatic lesions remains the preferred strategy for alleviating PTHrP-induced hypercalcemia, although unresectability predominates owing to metastatic disease at diagnosis. For unresectable, well-differentiated, and low-grade (G1/G2) neuroendocrine neoplasm liver metastases (NENs-LM), transarterial interventions constitute the preferred modality, including TAE, transarterial chemoembolization (TACE), and transarterial radioembolization. Current evidence demonstrates that TAE and TACE have comparable efficacy in the treatment of low-grade NENs-LM. TAE offers a superior response rate while avoiding chemotherapy-related adverse events related to TACE. Partial control of tumor progression and hypercalcemia via TACE, which has been documented in the literature (18, 39), typically results in a transient reduction of serum PTHrP after cytoreduction, followed by a rebound elevation during progression. Furthermore, ablative techniques (radiofrequency/microwave/cryoablation) provide supplementary treatment alternatives.

#### 3.3.2 Medical therapy

Pharmacologic management for hypercalcemia comprises intravenous isotonic saline, bisphosphonates, and somatostatin analogs (SSAs). Isotonic saline serves to correct hypovolemia secondary to renal salt consumption and vomiting, and hinders the worsening of hypercalcemia due to impaired renal calcium clearance (40). Although this therapy is regarded as a foundational supportive care, its efficacy as monotherapy remains limited (27). Bisphosphonates exert an inhibitory effect on osteoclast-mediated calcium release (41), yet the reduction of serum calcium is transient.

SSTRs comprise five subtypes, which are expressed in the majority of NETs (42). Specifically, SSTR2 is primarily overexpressed in well-differentiated NETs (43, 44). SSAs play a vital role in controlling the hormonal symptoms, delaying the tumor progression (45), and providing the partial disease control while mitigating the hypercalcemia recurrence. As an effective therapy for managing symptoms and hormone secretion in GEP-NETs, SSAs have been demonstrated to achieve a symptomatic response in 71% of patients and a biochemical response in 51% of patients (46, 47).

In addition to counteracting hormone excess in functional PNETs, SSAs have demonstrated antiproliferative efficacy in advanced disease. Systematic reviews have indicated that octreotide LAR achieves a disease stabilization rate of 15-88% (48). The maximum anti-tumor effects occur in patients with hepatic tumor burden ≤10% or those undergoing primary resection, which are irrelevant to functional status, serum chromogranin A levels, performance status, or age (49).

Combination therapy such as octreotide LAR with everolimus seems to outperform everolimus as a monotherapy (50). Nevertheless, therapeutic escape diminishes the efficacy of SSAs within 6–18 months of initiation (51). Multi-receptor targeting of SSAs like pasireotide demonstrates potential for enhanced therapeutic effect (52).

In addition, chemotherapy combined with temozolomide and capecitabine (CAPTEM regimen) is conducive to short-term control of both disease progression and hypercalcemia (18).

#### 3.3.3 Peptide receptor radionuclide therapy

PRRT with ^177^Lu-DOTATATE can reduce tumor volume, prolong overall and progression-free survival, and decrease serum calcium. This suggests a potential benefit in patients with PTHrP-hypersecretion, despite the fact that the underlying mechanism has not yet been fully elucidated (53).

### 3.4 Calcium-lowering experience

Hypercalcemia correlates with tumor progression, rendering tumor growth control as the cornerstone of calcium management. We observed that increased dose of SSAs provided partial calcium reduction in recurrent hypercalcemic crises. Conventional calcium-lowering agents, including salmon calcitonin, bisphosphonates, and denosumab, resulted in suboptimal responses. Subsequently, intermittent renal replacement therapy demonstrated superior efficacy, and CRRT sessions ≥48 hours indicated the best calcium reduction.

### 3.5 Challenges in managing cardio-renal failure

The patient experienced acute renal function deterioration during the disease course (**Figure 4**). Review of the medical history revealed mildly elevated serum creatinine levels early in the illness, suggesting possible pre-existing chronic kidney disease. Tumor-induced hypercalcemia contributed to renal vasoconstriction, reduced renal perfusion, and a consequent decline in glomerular filtration rate (GFR). Furthermore, deposition of calcium salts the renal tissues directly induced tubular injury (19, 54–56). Additional factors potentially exacerbating renal impairment included repeated contrast-enhanced CT scans, TAE, and exposure to nephrotoxic agents.

The sequelae of persistent hypercalcemia and subsequent renal failure exerted profound effects on cardiac function. Chronic hypercalcemia can lead to deposition of calcium salts within the myocardium, coronary arteries, and cardiac valves, resulting in myocardial fibrosis, reduced compliance, which ultimately impairs both diastolic and systolic function (19, 57, 58). In the later stages, myocardial function was further compromised by volume overload resulting from water and sodium retention, as well as by the direct cardiotoxic effects of uremic toxins. Electrolyte disturbances associated with renal failure, such as hyperkalemia, disrupted myocardial electrophysiology. Concomitant activation of the renin-angiotensin system (RAS) increased cardiac afterload, while Anemia and malnutrition further diminished myocardial energy supply. Together, these factors synergistically precipitated cardiac failure. Given the presence of generalized edema, fatigue, and elevated PRO-BNP, etiological assessment of cardiac dysfunction suggested possible carcinoid heart disease, despite the non-supportive echocardiographic findings.

Escalation of octreotide LAR dose stabilized tumor growth. The potential benefits of chemotherapy (CAPTEM regimen) were anticipated but not pursued. Specifically, combined liver-kidney transplantation was recommended upon the onset of renal failure, but the patient declined both chemotherapy and transplantation. Therefore, the rapid development of refractory heart failure precluded opportunities for further treatment.

### 3.6 Analysis of dyspnea etiology

Paroxysmal dyspnea rendered the patient critically ill. After excluding acute heart failure, bronchial asthma, diabetic ketoacidosis, and psychological factors, symptoms resolved following continuous somatostatin infusion. This suggested that somatostatin may inhibit hormones secreted by tumors, which warranted further investigation.

## 4 Conclusion

Hypercalcemia, a rare manifestation of F-PNETs, is typically mediated by PTHrP hypersecretion, and its progression often reflects tumor advancement. In acute management, rapid calcium reduction is prioritized. Based on our experience, CRRT achieves the most rapid correction and the longest maintenance of normocalcemia. Long-term control requires cytoreduction and suppression of PTHrP release. Thus, early diagnosis and individualized therapeutic strategies are essential to improve the survival and prognosis of patients.

## Data Availability

The original contributions presented in the study are included in the article/**Supplementary Material**. Further inquiries can be directed to the corresponding author.
