# Supplementary material for: Functional pancreatic neuroendocrine tumors predominantly presenting with hypercalcemia: a case report
Source: Front Oncol. 2026 Jan 5;15:1693096. doi: 10.3389/fonc.2025.1693096 (PMC12812571; doi:10.3389/fonc.2025.1693096)
Supplement: Supplementary file 1 [file DataSheet1.pdf]

Serum calcium, PTH and phosphorus levels

Date	Ca2+(mmol/L) 2.11-2.52 mmol/L	PTH（pg/ml） 15.00-65.00 pg/mL	P （mmol/L） 0.85-1.51 mmol/L
2023/3/14	3.49		0.85
2023/3/23	4.08		
2023/3/26	4.85		
2023/3/27	3.83		
2023/3/27	3.45		
2023/3/28	2.99		
2023/3/28	3.43		
2023/3/29	2.73		
2023/3/30	2.49		
2023/3/31	2.22		
2023/4/1	2.06		
2023/4/5	1.85		
2023/4/8	1.70		
2023/4/11	1.67		
2023/4/12	2.03		
2023/5/13	2.52		
2023/5/17	2.28		
2023/6/22	2.51		1.13
2023/7/25	2.56		
2023/8/23	3.69		
2023/8/23	3.31		
2023/8/24	3.20		
2023/8/25	2.90		
2023/8/29	2.77		
2023/8/31	2.70		
2023/9/4	2.47		
2023/9/27	2.61		
2023/9/28	2.74		1.12
2023/10/17	2.33		
2023/10/26	2.62		
2023/10/27	2.52		
2023/10/30	2.92	101.5	
2023/11/30	2.49	31.34	
2023/12/28	2.99	6.25	1.13
2023/12/29	3.00		
2024/1/1	2.87		
2024/1/12	3.15		
2024/1/22	3.78	5.31	
2024/1/23	3.67		
2024/1/25	3.81		
2024/1/29	3.40		
2024/2/1	3.67		
2024/2/4	3.64		
2024/2/5	3.31		
2024/2/6	3.48		
2024/2/7	3.12		
2024/2/9	3.17		
2024/2/11	3.23		
2024/2/13	3.16		
2024/2/14	3.33		
2024/2/15	3.39		
2024/2/15	2.79		
2024/2/18	3.86		

2024/2/19	3.99		
2024/2/20	3.26		
2024/2/20	2.88		
2024/2/21	3.64		
2024/2/22	3.19		
2024/2/22	2.85		
2024/2/23	2.79		
2024/2/23	2.60		
2024/2/24	3.29		
2024/2/24	2.75		
2024/2/25	3.03		
2024/2/26	3.22		
2024/2/28	2.99		
2024/2/29	3.22		
2024/3/19	2.93	9.68	
2024/3/20	2.87		
2024/3/21	2.85		
2024/3/22	2.52		0.97
2024/3/23	2.47		
2024/3/25	2.36		
2024/3/27	2.45		
2024/3/29	2.22		
2024/3/31	2.08		
2024/4/8	1.98	396.1	
2024/4/19	1.75		
2024/4/29	1.65		
2024/5/9	1.86		
2024/5/20	2.16		
2024/5/30	2.12		
2024/6/11	2.21	446	
2024/6/20	2.58		1.41
2024/7/1	2.39		
2024/7/11	3.28		
2024/7/12	3.46		
2024/7/14	3.92		
2024/7/15	3.88	7.22	
2024/7/16	4.15		
2024/7/17	3.96		
2024/7/18	4.08		
2024/7/19	4.17		
2024/7/20	3.83		
2024/7/22	2.40		
2024/7/23	2.35		
2024/7/24	2.10		
2024/8/6	1.30		
2024/8/16	1.00		
2024/8/26	1.16	699.1	
2024/9/5	1.57	646.3	
2024/9/15	1.94		
2024/9/16	1.86		
2024/9/25	2.29	480.8	1.04
2024/9/26	2.08		
2024/9/27	2.12		
2024/9/28	2.06		
2024/10/8	2.27		
2024/10/18	2.34		
2024/10/28	2.56	39.9	
2024/10/29	2.69		

2024/10/30	2.56		
2024/10/31	2.54		
2024/11/7	2.83		
2024/11/8	2.67		
2024/11/11	2.34		
2024/11/18	2.83		
2024/11/28	3.23	6.92	
2024/11/29	2.93		
2024/11/30	3.00		
2024/12/1	2.77		
2024/12/2	2.55		
2024/12/3	2.22		
2024/12/9	2.22		0.89
2024/12/11	2.15		
2024/12/20	2.28		
2025/1/10	2.72		
2025/1/20	2.12		
2025/2/5	1.38		
2025/2/6	1.36		
2025/2/8	1.28		
2025/2/20	1.66		
2025/3/3	1.61		
2025/3/5	1.55		
2025/3/6	1.69		
2025/3/7	1.78		
2025/3/9	1.82		
2025/3/25	2.29		
2025/3/27	2.27		1.7
2025/3/30	2.68		
2025/4/2	2.68		
2025/4/11	2.98		
2025/4/12	2.68		
2025/4/13	2.61		
2025/4/14	2.22		
2025/4/17	2.12		
2025/4/20	2.09		
2025/4/23	2.07		
2025/4/28	1.99		
2025/5/13	3.19		
2025/5/17	2.50		
2025/5/25	2.00		
2025/5/26	2.22		
2025/5/27	2.24		
2025/5/28	2.07		
2025/5/29	1.95		
2025/6/1	1.73		
2025/6/3	1.49		
2025/6/10	1.70		
2025/6/13	1.65		
2025/6/16	1.46		1.82

Calcitonin (CT)

Date	CT（pg/ml）<9.52 pg/mL
2023/3/28	> 2000
2023/10/30	511.7

2023/11/30	515.6
2023/12/28	1008
2024.01.22	> 2000
2024/3/19	> 2000
2024/4/8	881.7
2024/6/11	432.2
2024/7/15	> 2000
2024/8/26	305
2024/9/5	527.9
2024/9/25	1222
2024/10/28	1958
2024/11/28	2000

Liver function

Date	ALT(U/L) 5-40 U/L	AST(U/L) 8-40 U/L	ALP(U/L) 45-125 U/L	GGT(U/L) 11-50 U/L	TBIL(umol/L) 3.4-20.5umol/L
2023/3/14	37	21	321	390	7.8
2023/6/22	14	23	97	142	5.2
2023/9/27	13	15	137	208	7.2
2023/12/28	16	23	111	140	10.8
2024/3/19	14	17	351	333	10.9
2024/6/11	11	20	213	210	5.4
2024/9/15	21	27	386	291	9.1
2024/12/16	18	35	487	324	8.9
2025/3/25	20	27	474	364	12
2025/6/16	18	158	809	343	39.8

Renal function

Date	CREA(umol/L) 62-115umol/L	eGFR(ml/min) 56-122ml/min
2023/3/14	153	41
2023/6/22	201	29
2023/9/27	210	28
2023/12/28	185	32
2024/3/19	137	47
2024/4/8	207	28
2024/6/11	288	19
2024/8/16	235	24
2024/8/26	258	22
2024/9/15	252	22
2024/9/25	231	25
2024/12/16	238	24
2025/3/25	272	20
2025/6/16	410	12
